# Global temporal changes in the proportion of children with advanced disease at the start of combination antiretroviral therapy in an era of changing criteria for treatment initiation

**DOI:** 10.1002/jia2.25200

**Published:** 2018-11-22

**Authors:** Klea Panayidou, Mary‐Ann Davies, Nanina Anderegg, Matthias Egger, The IeDEA, COHERE, PHACS and IMPAACT 219C Collaborations Writing Group

**Affiliations:** ^1^ Institute of Social and Preventive Medicine University of Bern Bern Switzerland; ^2^ Centre for Infectious Disease Epidemiology and Research School of Public Health and Family Medicine University of Cape Town Cape Town South Africa

**Keywords:** antiretroviral therapy, advanced HIV disease, CD4 cell count, WHO guidelines, sub‐Saharan Africa, North America, Caribbean, Central and South America, Europe, Asia

## Abstract

**Introduction:**

The CD4 cell count and percent at initiation of combination antiretroviral therapy (cART) are measures of advanced HIV disease and thus are important indicators of programme performance for children living with HIV. In particular, World Health Organization (WHO) 2017 guidelines on advanced HIV disease noted that >80% of children aged <5 years started cART with WHO Stage 3 or 4 disease or severe immune suppression. We compared temporal trends in CD4 measures at cART start in children from low‐, middle‐ and high‐income countries, and examined the effect of WHO treatment initiation guidelines on reducing the proportion of children initiating cART with advanced disease.

**Methods:**

We included children aged <16 years from the International Epidemiology Databases to Evaluate acquired immunodeficiency syndrome (AIDS) (IeDEA) Collaboration (Caribbean, Central and South America, Asia‐Pacific, and West, Central, East and Southern Africa), the Collaboration of Observational HIV Epidemiological Research in Europe (COHERE), the North American Pediatric HIV/AIDS Cohort Study (PHACS) and International Maternal Pediatric Adolescent AIDS Clinical Trials (IMPAACT) 219C study. Severe immunodeficiency was defined using WHO guidelines. We used generalized weighted additive mixed effect models to analyse temporal trends in CD4 measurements and piecewise regression to examine the impact of 2006 and 2010 WHO cART initiation guidelines.

**Results:**

We included 52,153 children from fourteen low‐, eight lower middle‐, five upper middle‐ and five high‐income countries. From 2004 to 2013, the estimated percentage of children starting cART with severe immunodeficiency declined from 70% to 42% (low‐income), 67% to 64% (lower middle‐income) and 61% to 43% (upper middle‐income countries). In high‐income countries, severe immunodeficiency at cART initiation declined from 45% (1996) to 14% (2012). There were annual decreases in the percentage of children with severe immunodeficiency at cART initiation after the WHO guidelines revisions in 2006 (low‐, lower middle‐ and upper middle‐income countries) and 2010 (all countries).

**Conclusions:**

By 2013, less than half of children initiating cART had severe immunodeficiency worldwide. WHO treatment initiation guidelines have contributed to reducing the proportion of children and adolescents starting cART with advanced disease. However, considerable global inequity remains, in 2013, >40% of children in low‐ and middle‐income countries started cART with severe immunodeficiency compared to <20% in high‐income countries.

## 1 Introduction

World Health Organization (WHO) guidelines on initiating combination antiretroviral therapy (cART) in children have expanded paediatric cART eligibility. The initial WHO guidelines (2002, revised in 2006) established CD4 thresholds for cART initiation across all paediatric age groups 1. In 2010, immediate cART initiation regardless of immunological or clinical thresholds was recommended for children aged <2 years 2 and expanded in 2013 to all children <5 years 3 and in 2016 to all adults and children living with HIV 4. These modifications were largely predicated by the rapid HIV disease progression and high morbidity and mortality in infants and children 5, 6, 7, together with evidence of lower mortality (particularly in infants <3 months of age) 8 as well as better growth, immunological and morbidity outcomes and less chronic organ system disease associated with early cART initiation 9, 10, 11, 12, 13, 14, 15, 16, 17, 18, 19. An additional motivation for recommending earlier cART in children was to address the lag in paediatric cART coverage 20.

Serial monitoring of CD4 at cART initiation is important for programme evaluation, indicating the extent to which guideline changes are successfully implemented so that children initiate cART before onset of advanced HIV disease. An analysis from sub‐Saharan Africa, Asia, North America and Latin America from 2004 to 2010 demonstrated reductions in the proportions of children initiating cART with severe immunodeficiency, but even in 2010 the vast majority of children in low‐ and middle‐income countries continued to start cART late 21. The ongoing high burden of advanced HIV disease was a key motivation for the recent WHO 2017 guidelines on managing advanced disease 22. We analysed CD4 measures at cART initiation from an international collaboration of treatment programmes in sub‐Saharan Africa, Asia, Europe, Central and South America, and North America from 1996 to 2013 in order to assess changes in the proportion of children starting cART with advanced HIV disease and the impact of WHO treatment initiation guidelines on this proportion.

## 2 Methods

### 2.1 Data sources

Data were collated from four major cohort research networks: the International Epidemiology Databases to Evaluate AIDS (IeDEA); the Collaboration of Observational HIV Epidemiological Research Europe (COHERE); the Adolescent Master Protocol (AMP) study of the Pediatric HIV/AIDS Cohort Study (PHACS) network; and the 219C Long‐Term Follow‐Up Study of the International Maternal Pediatric Adolescent AIDS Clinical Trials (IMPAACT) network. IeDEA is a global consortium with regional centres that pool clinical and epidemiological data on individuals living with HIV; regions collecting paediatric data are Caribbean/Central and South America, Asia‐Pacific, East Africa, West Africa, Central Africa and Southern Africa 23. COHERE is a collaboration of European HIV cohorts that conducts epidemiological research on the prognosis of people living with HIV across Europe 24. IMPAACT 219C and PHACS AMP are US‐based prospective cohort studies designed to evaluate the impact of HIV infection and cART on youth. New enrolment in recent years is limited 25, 26, 27. Pooling of data and their use in collaborative analyses were approved by local institutional review boards. For the present study, regional centres sent de‐identified data to the University of Bern, Switzerland, for cleaning and analysis.

### 2.2 Inclusion criteria and definitions

All patients living with HIV aged <16 years at cART start (irrespective of likely mode of infection) were included in descriptive analyses if they had documented sex and cART start date after 1994, were treatment‐naïve at cohort entry (except for exposure to antiretrovirals for prevention of mother‐to‐child transmission (PMTCT)) or had CD4 measured at first cART start if not naïve at cohort entry. For further analyses, we excluded data from low‐ or middle‐income countries before widespread cART rollout began in 2002 and from countries that contributed <50 patients with a CD4 measurement at cART start. We also excluded data on children who started therapy in a year and country for which <10 children with CD4 measures were reported as well as data from the last calendar year in countries where no CD4 measures were available after May of that calendar year. cART was typically defined by participating cohorts as a regimen of ≥3 antiretroviral drugs from ≥2 drug classes. The baseline CD4 value was defined as the value nearest the cART start date within −6 to +1 months of start. Countries were grouped according to the World Bank classification of annual Gross National Income per capita 2013 as low‐income (LIC, ≤US$1045), lower middle‐income (LMIC, US$1046‐4125), upper middle‐income (UMIC, US$4126 to 12,745) and high‐income (HIC, ≥US$12,746). We used the 2013 classification as this was the most recent year for which data was included 28. Age groups at cART initiation were less than twelve months, twelve to thirty‐five months, thirty‐six to fifty‐nine months, five to eleven years and twelve to fifteen years. Severe immunodeficiency was defined according to WHO as CD4% <25% (age <12 months), <20% (12 to 35 months), <15% (36 to 59 months) and CD4 count <200 cells/μL or CD4% <15% (≥5 years) 29.

### 2.3 Multiple imputation of missing CD4 measurements

We imputed missing CD4 measures at cART initiation from 1996 (HIC) and 2002 (LIC, LMIC and UMIC) onwards for countries and calendar years. We imputed the arcsine square root of CD4% and square root of CD4 cell count simultaneously using chained equations and predictive mean matching, adjusting for country and year of cART start, stratifying by sex, age, income group and cohort. We generated 50 imputed data sets and combined these using Rubin's rule 30.

### 2.4 Analysis of temporal trends in CD4 measurements

We used generalized additive mixed models to analyse temporal trends in CD4 measures by sex, age and country income group. We assessed three key outcomes at cART initiation: the proportion of children with severe immunodeficiency; median CD4% (aged <5 years); and median CD4 count (aged ≥5 years). Sex, age group and income group and their interactions were entered as fixed effects and country as a random intercept. Yearly trends were smoothed by sex, age group and income group. Data were aggregated by calendar year (three to sixteen years, depending on country), country (thirty‐two countries), sex and age group prior to analysis: each combination of these factors corresponded to a cell in the analysis. For each cell, we calculated the number of children with severe immunodeficiency and the median CD4% and count. Each cell was entered in the model with a two‐part weight. The first part incorporated the precision of the aggregated values for each cell into the model and corresponded to the number of observations contained in a cell, divided by the average number of observations in all cells in the same income group. The second part corresponded to the ratio of the number of patients that were newly enrolled in that cohort for that year and the number of patients that started cART in that country during that year 31. The weights of the second part were also normalized by country income group. We used the data set with imputed data for the main analysis. In sensitivity analyses, we fitted the model restricted to the subset with complete data.

### 2.5 Influence of WHO guidelines on the proportion starting cART with advanced disease

From 2002 to 2013, there were two major changes in WHO paediatric cART initiation guidelines. Before 2006, WHO guidelines for initiation of cART in children were included in the adult guidelines; children were cART eligible with WHO Stage 3 disease (AIDS; note Stage 4 disease not defined at that time) or Stage 2 disease if CD4% <20% (age <18 months) or <15% (age >18 months) 32. In 2006, WHO released revised stand‐alone guidelines for children, based on a public health approach 1. In the 2006 guidelines, WHO cART eligibility criteria were: WHO Clinical Stages 3/4, CD4 < 1500 cells/μL or 25% (age <12 months), CD4 < 750 cells/μL or 20% (12 to 35 months), CD4 < 350 cells/μL or 15% (36 to 59 months) and CD4 ≤ 200 cells/μL (≥5 years) 1. In 2010, WHO recommended cART regardless of immunologic or clinical thresholds for children <2 years, children with WHO Clinical Stages 3/4, CD4 < 750 cells/μL or 25% (age two to five years) and CD4 ≤ 350 cells/μL (age ≥5 years) 2. We performed segmented (piecewise) linear regression to test whether the guideline changes affected the speed of decline in the proportion of children with severe immunodeficiency at cART initiation. Segments were calendar periods 2002 to 2005, 2006 to 2009 and 2010 to 2013, assuming change points at 2006 and 2010 1, 2. We used a generalized linear mixed effects model with income groups as fixed effects and countries as random effects. The main slope was the middle period (2006 to 2009). The variability in the country effect was expressed by a different intercept, allowing parallel deviations from the average income group and random slopes, accounting for slower or faster than average decreases. The model allowed different slopes for different periods but forced the line segments for the three periods to be continuous at the change points, and the change in slopes between periods to be the same for all countries within each income group. We estimated confidence intervals (CI) to determine whether slopes in 2002 to 2005 and 2010 to 2013 differed from the reference slope (2006 to 2009).

The technical appendix provides further details on the multiple imputation and smoothing (see Appendix 1). Analyses were conducted in R 3.1.0 (R Core Team, Vienna, Austria).

## 3 Results

### 3.1 Descriptive analyses

**Figure 1:**
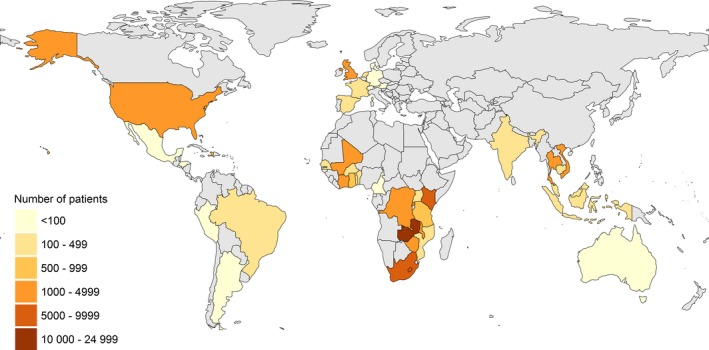
**Map of countries contributing patients to the collaborative analysis**

Data from 67,486 children from 44 countries <16 years old at cART start were submitted to the data centre (Figure S1). We excluded 13,383 participants who did not meet the inclusion criteria, mostly because they were not treatment‐naïve at cohort entry; hence, 54,103 children from 41 countries were included in descriptive analyses (Figure 1). We excluded 1950 children from further analyses using multiple imputation (nine countries) either because their CD4 measures were before 1996 (HIC) or 2002 (LIC, LMIC and UMIC) (268 children), or from calendar years with <10 children in that country with CD4 measures (1544 children) or with no measure after May of the last calendar year for the relevant country (138 children) (Figure S1). Data typically spanned the years 2003 to 2013 in low‐ and middle‐income countries and 1997 to 2012 in HIC; in the USA, data were only available for 1996 to 2006, as all participants initiated cART before 2007 (Table 1). The median year of cART initiation ranged from 1998 in the US to 2012 in Mozambique. The median (interquartile range (IQR)) age of children starting cART was 6 years (3 to 10) in LIC, 5 (2 to 10) in LMIC, 6 (2 to 10) in UMIC and 6 (2 to 10) in HIC. Median CD4 cell counts/percentages at cART initiation by country and sex are shown in Table 2. The median CD4 count at cART start for children ≥5 years old was 250 cells/μL (106 to 416) in LIC, 258 (118 to 446) (LMIC), 189 (57 to 349) (UMIC) and 348 (179 to 601) (HIC). Similar patterns were evident for CD4% in children <5 years (Table 2). The overall percentage (95%CI) of children starting cART with severe immunodeficiency was 48% (47% to 49%) (LIC), 52% (51% to 53%) (LMIC), 57% (56% to 58%) (UMIC) and 31% (30% to 33%) (HIC).

**Table 1:** Characteristics at cART initiation of 54,103 children starting cART by country income group

Country	Number of patients		Median age in years		Median calendar year of starting cART		Calendar year range of data	Calendar year range of data used in analysis
	Female	Male	Female	Male	Female	Male		
Low‐income								
Benin	103	120	4	4	2008	2009	2002 to 2012	2004 to 2011
Burkina Faso	196	176	7	6	2008	2009	2001 to 2012	2003 to 2012
Cambodia	191	196	6	6	2007	2007	2004 to 2012	2005 to 2010
Congo, DR	529	524	6	6	2009	2009	2004 to 2013	2004 to 2013
Haiti	373	314	6	7	2009	2008	2003 to 2013	2003 to 2012
Kenya	4227	4199	6	6	2010	2010	2002 to 2012	2003 to 2012
Malawi	2113	2072	6	6	2009	2009	2000 to 2013	2005 to 2010
Mali	465	659	4	4	2007	2007	2001 to 2012	2002 to 2012
Mozambique	217	224	2	2	2012	2012	2005 to 2014	2007 to 2013
Rwanda	506	497	9	8	2008	2008	2000 to 2013	2004 to 2013
Tanzania, UR	398	378	7	6	2009	2009	2005 to 2013	2005 to 2012
Togo	155	154	4	4	2011	2011	2005 to 2012	2010 to 2012
Uganda	239	252	4	5	2010	2010	2002 to 2012	2006 to 2011
Zimbabwe	1535	1537	9	9	2011	2011	1999 to 2014	2005 to 2013
Overall (IQR)	11,247	11,302	6 (3 to 11)	6 (3 to 10)	2010 (2007 to 2011)	2009 (2007 to 2011)	1999 to 2014	2002 to 2013
Lower middle‐income								
Cameroon	4	1	12	15	2006	2007	2003 to 2008	Excluded
Cote d'Ivoire	973	1030	6	6	2007	2007	2001 to 2012	2004 to 2012
Ghana	249	276	6	5	2008	2009	2001 to 2012	2004 to 2012
Honduras	16	13	3	5	2005	2005	2002 to 2009	Excluded
India	49	68	9	8	2008	2008	2001 to 2012	2005 to 2010
Indonesia	103	111	3	2	2009	2008	2005 to 2013	2005 to 2012
Lesotho	355	381	6	6	2011	2010	2003 to 2014	2006 to 2013
Senegal	150	196	6	5	2009	2008	2000 to 2012	2005 to 2011
Vietnam	493	621	4	4	2009	2009	2005 to 2013	2005 to 2012
Zambia	7315	7090	6	5	2009	2009	2003 to 2011	2004 to 2011
Overall (IQR)	9707	9787	6 (2 to 10)	5 (2 to 9)	2009 (2007 to 2010)	2009 (2007 to 2010)	2000 to 2014	2004 to 2013
Upper middle‐income								
Argentina	5	5	7	6	2002	2009	2001 to 2010	Excluded
Brazil	212	200	4	5	2003	2003	1997 to 2012	2002 to 2012
Malaysia	119	130	5	4	2008	2008	2001 to 2013	2003 to 2011
Mexico	1	1	11	3	2007	1999	1998 to 2006	Excluded
Peru	54	44	6	3	2006	2006	2002 to 2014	2004 to 2007
South Africa	3328	3207	5	5	2009	2009	2000 to 2013	2002 to 2013
Thailand	686	595	8	8	2006	2006	2000 to 2013	2002 to 2012
Overall (IQR)	4405	4182	6 (2 to 10)	6 (2 to 9)	2009 (2006 to 2011)	2009 (2006 to 2011)	1997 to 2014	2002 to 2013
High‐income								
Australia	1	0	11	‐	2000	‐	1999 to 1999	Excluded
Austria	1	0	16	‐	2001	‐	2001 to 2001	Excluded
Denmark	31	17	8	7	2003	2003	1997 to 2012	Excluded
France	115	92	1	1	2005	2004	1996 to 2013	1997 to 2008
Germany	11	22	6	9	2004	2005	1998 to 2011	Excluded
Netherlands	114	115	3	4	2004	2004	1997 to 2013	1997 to 2012
Spain	181	159	2	2	2005	2005	1996 to 2013	1998 to 2011
Switzerland	1	0	16	‐	1999	‐	1999 to 1999	Excluded
United Kingdom	601	590	7	6	2005	2004	1996 to 2013	1997 to 2012
United States	741	681	7	6	1998	1998	1995 to 2010	1996 to 2006
Overall (IQR)	1797	1676	6 (2 to 10)	6 (2 to 10)	2002 (1998 to 2006)	2001 (1998 to 2006)	1995 to 2013	1996 to 2012

cART, combination antiretroviral therapy; IQR, interquartile range.

**Table 2:** CD4 cell count and CD4% at the start of cART and the percentage of children starting cART with severe immunodeficiency by country income group. In the imputed analysis, 52,153 children were included and in the complete case analysis 34,363

	Percentage of children missing both CD4 count and CD4% measurements		Median CD4 cell count at start of cART in cells/μL of children 5 years and older				Median CD4% at start of cART in cells/μL of children younger than 5 years				Percentage of children starting cART with severe immunodeficiency			
			Complete case		Imputed data		Complete case		Imputed data		Complete case		Imputed data	
	Female	Male	Female	Male	Female	Male	Female	Male	Female	Male	Female	Male	Female	Male
Low‐income														
Benin	15%	19%	161	146	159	146	20	24	15	17	67%	58%	65%	54%
Burkina Faso	27%	28%	314	284	314	280	12	13	12	14	52%	54%	53%	54%
Cambodia	4%	5%	180	213	162	195	12	11	12	11	70%	68%	68%	63%
Congo, DR	17%	18%	302	217	302	218	‐	‐	‐	‐	37%	46%	38%	46%
Haiti	9%	5%	255	248	255	252	18	18	18	18	46%	48%	48%	49%
Kenya	19%	18%	281	277	281	278	15	14	16	15	45%	47%	46%	49%
Malawi	76%	79%	249	262	249	259	16	14	15	14	46%	53%	47%	54%
Mali	8%	6%	210	169	214	169	16	15	15	13	59%	62%	61%	66%
Mozambique	44%	47%	369	427	368	402	8	16	14	14	35%	53%	59%	69%
Rwanda	28%	26%	279	283	277	283	‐	‐	‐	‐	33%	33%	37%	36%
Tanzania, UR	39%	40%	160	122	151	117	40	45	28	13	65%	67%	49%	67%
Togo	36%	34%	377	366	386	375	‐	‐	16	15	40%	38%	51%	49%
Uganda	24%	25%	151	150	151	150	15	14	16	14	64%	68%	64%	68%
Zimbabwe	29%	31%	233	217	231	214	15	14	15	14	45%	49%	48%	54%
Overall (IQR)	30%	30%	254 (119 to 418)	243 (94 to 411)	253 (120 to 411)	244 (99 to 410)	15 (10 to 22)	14 (10 to 20)	16 (11 to 22)	14 (10 to 20)	46%	50%	48%	53%
Lower middle‐income														
Cote d'Ivoire	22%	25%	279	293	284	311	14	13	14	13	51%	56%	50%	52%
Ghana	29%	24%	257	237	257	231	‐	‐	12	14	46%	43%	57%	60%
India	7%	14%	227	193	227	193	10	14	10	14	48%	63%	48%	63%
Indonesia	13%	10%	57	63	49	63	12	10	12	7	81%	82%	82%	83%
Lesotho	31%	32%	270	262	256	251	22	16	19	16	44%	47%	46%	50%
Senegal	37%	30%	221	264	213	267	16	14	14	14	49%	51%	56%	58%
Vietnam	10%	11%	164	117	163	119	16	14	15	13	62%	67%	62%	67%
Zambia	23%	21%	265	259	264	261	17	16	16	16	49%	52%	50%	54%
Overall (IQR)	22%	21%	261 (120 to 447)	254 (115 to 444)	262 (121 to 447)	259 (116 to 449)	16 (11 to 23)	15 (10 to 21)	16 (11 to 22)	15 (10 to 21)	50%	54%	51%	55%
Upper middle‐income														
Brazil	19%	15%	444	299	443	297	19	19	19	19	33%	40%	32%	42%
Malaysia	20%	17%	77	177	77	175	17	16	16	14	73%	64%	72%	65%
Peru	22%	23%	267	68	270	205	‐	‐	16	20	48%	80%	46%	52%
South Africa	29%	30%	220	218	223	225	17	16	17	16	54%	56%	54%	55%
Thailand	14%	13%	112	67	112	64	14	13	13	13	64%	72%	64%	72%
Overall (IQR)	26%	27%	193 (70 to 353)	183 (46 to 342)	200 (75 to 361)	196 (52 to 356)	17 (11 to 23)	16 (11 to 23)	17 (11 to 23)	15 (10 to 23)	56%	58%	55%	57%
High‐income														
France	9%	8%	283	416	310	416	36	34	35	35	29%	28%	26%	27%
Netherlands	18%	18%	310	320	310	320	24	18	22	19	36%	39%	32%	40%
Spain	21%	27%	323	389	286	368	29	25	29	26	35%	38%	32%	33%
United Kingdom	17%	16%	272	253	270	251	20	17	20	17	39%	48%	40%	48%
United States	1%	1%	484	441	484	441	31	28	31	28	18%	24%	18%	24%
Overall (IQR)	10%	11%	350 (206 to 608)	342 (154 to 597)	341 (196 to 590)	331 (152 to 590)	28 (17 to 38)	24 (14 to 33)	27 (17 to 37)	24 (14 to 33)	28%	35%	28%	35%

cART, combination antiretroviral therapy; IQR, interquartile range.

### 3.2 Analysis of temporal trends in CD4 measurements

**Figure 2:**
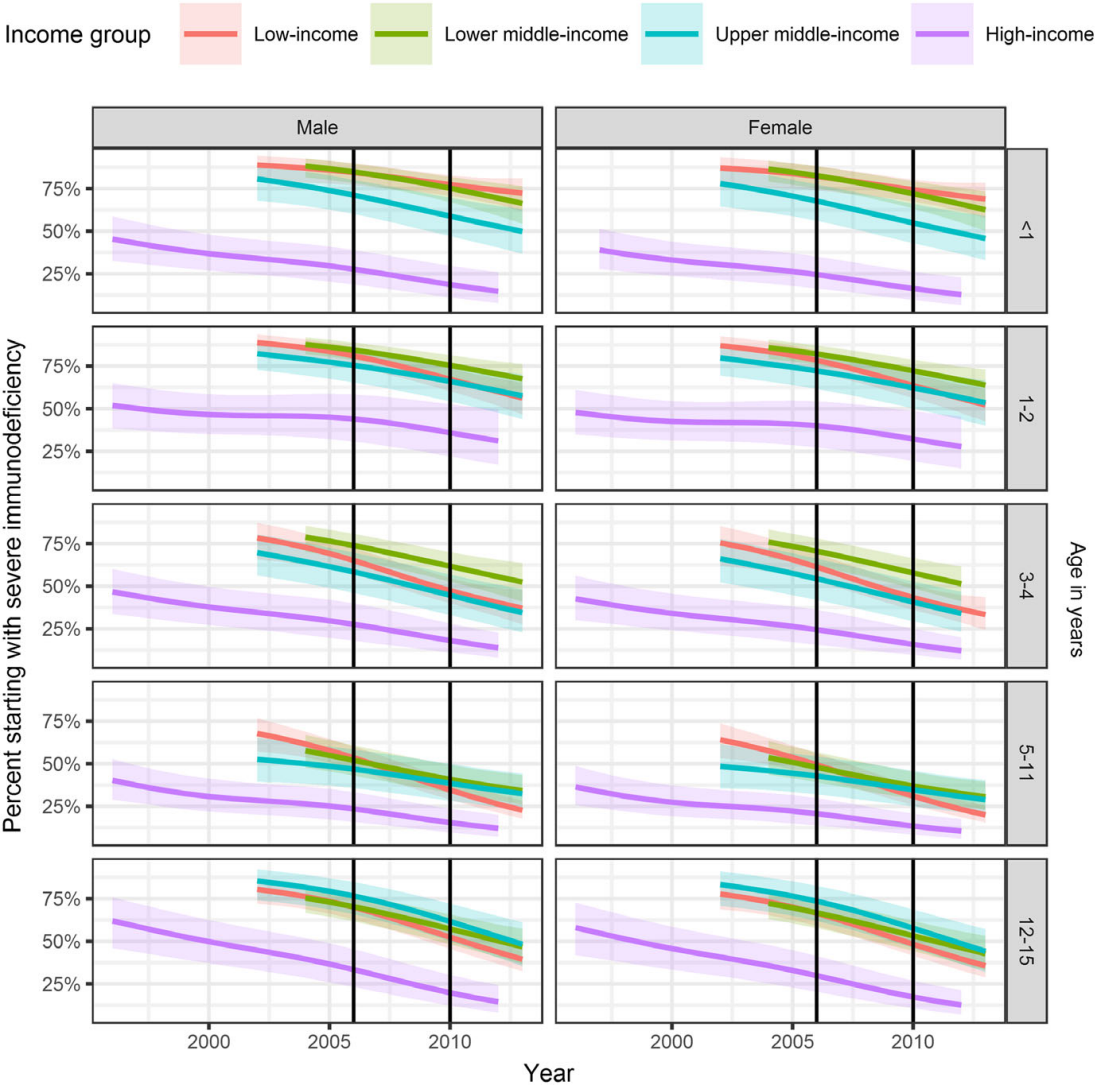
**Severe immunodeficiency at the start of cART by age, sex and country income groups (colours)** Severe immunodeficiency was defined according to WHO as CD4% <25% (age <12 months), <20% (12 to 35 months), <15% (36 to 59 months) and CD4 count <200 cells/μL or CD4% <15% (≥5 years). Results from generalized additive mixed effects models based on 52,153 children after imputation of missing data. 95% CIs are shown as shaded areas. Vertical lines indicate the changes in WHO guidelines on when to start cART. Age groups are shown along the right edge. Note that no data available for the analysis for 2003 in LMIC and after 2012 for HIC; hence, the periods shown on the graph differ slightly by country income group. cART, combination antiretroviral therapy; WHO, World Health Organization; LMIC, lower middle‐income country; HIC, high‐income country.

**Figure 3:**
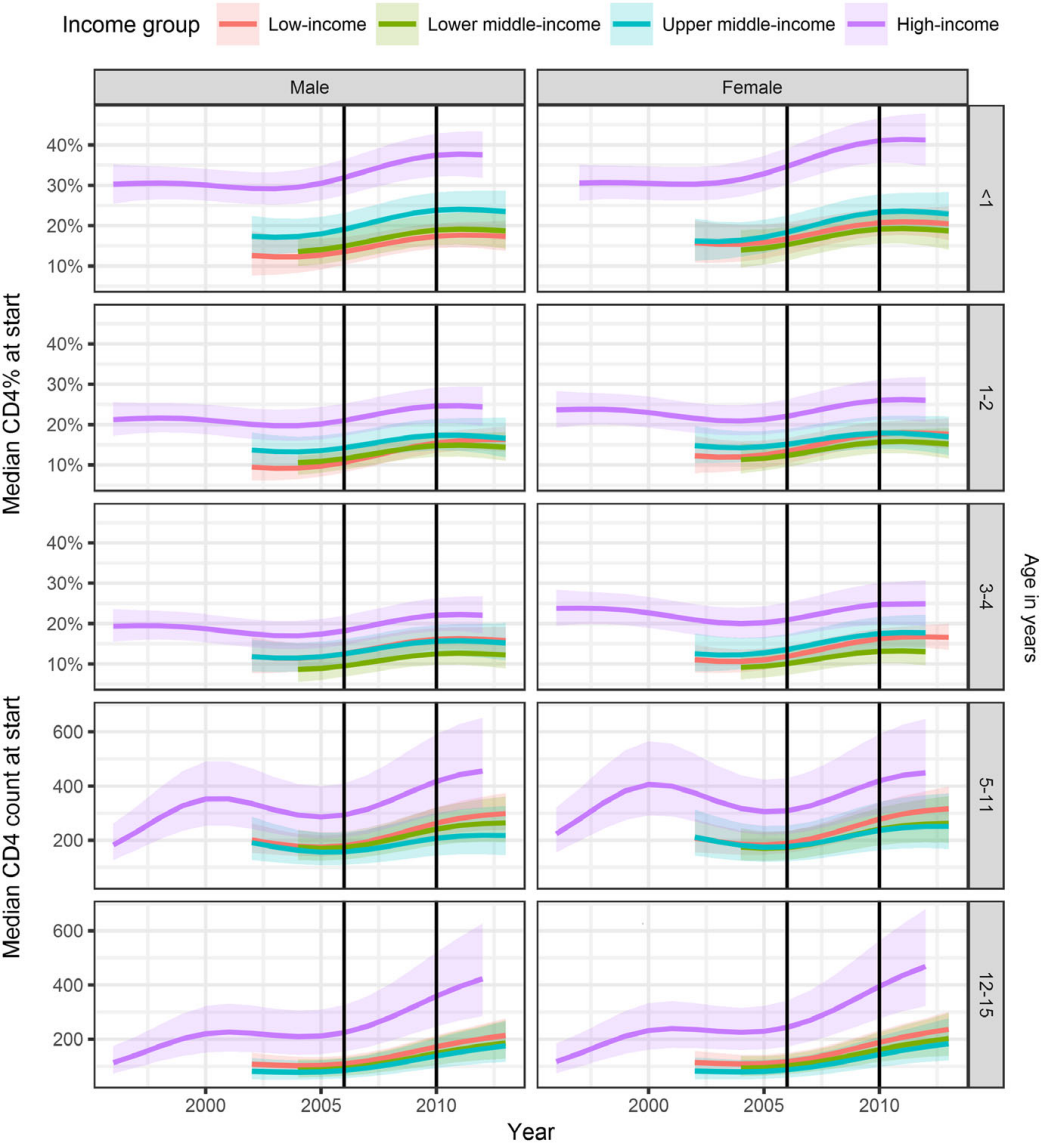
**Median CD4 cell count in children aged 5 years or older and median CD4% in children below 5 years of age at the start of cART by age, sex and income group (colours)** Results from generalized additive mixed effects models based on 52,153 children after imputation of missing data. 95% CIs are shown as shaded areas. Vertical lines indicate the changes in WHO guidelines on when to start cART. Age groups are shown along the right edge. cART, combination antiretroviral therapy; WHO, World Health Organization.

Further analyses were based on 52,153 children, among whom CD4 measurements were imputed for 17,790 patients. (Table S1). Figure 2 shows modelled temporal trends in the prevalence of severe immunodeficiency at cART initiation. Figure 3 shows corresponding trends in median CD4 counts (children aged ≥5 years) or CD4% (children <5 years). In LIC, the estimated percentage of children starting cART with severe immunodeficiency declined from 70% in 2004 to 42% in 2013. Corresponding figures were 67% to 46% (LMIC), 61% to 43% (UMIC) and 45% to 14% (HIC, 1996 to 2012). Of note, in LIC among children aged <1 year, there was almost no decline in the proportion starting cART with severe immunodeficiency.

In LIC, the median CD4 cell count at cART initiation in children aged ≥5 years increased by 67% during 2004 to 2013, (162 to 271 cells/μL). Corresponding increases were 55% (151 to 234 cells/μL) (LMIC), 35% (164 to 222 cells/μL) (UMIC) and 144% (185 to 452 cells/μL) (HIC, 1996 to 2012). In children aged <5 years, median CD4% increased during 2004 to 2013 from 11% to 17% in LIC, 10% to 16% in LMIC, 14% to 20% in UMIC and 22% to 29% in HIC (1996 to 2012) (Figure 3). Results of complete case analysis were similar (Figures S2 and S3).

Temporal trends in median age at cART initiation varied among country income groups (Figure S4). There was no clear trend in median age at cART initiation in LMIC, while there was a decrease from 2004 to 2013 in LIC (6.2 to 4.6 years) and an increase in UMIC (4.5 to 7.8 years) as well as HIC (2.1 (1996) to 7.4 years (2012)).

### 3.3 Influence of WHO guidelines on the proportion starting cART with advanced disease

There was a significant decrease in the percentage of children starting cART with severe immunodeficiency in at least one period in all country income groups (Table 3A, Table S2A). The annual decrease in the proportion of children with severe immunodeficiency at cART initiation between 2006 and 2009 was −3.0% (95% CI: −4.1 to −1.9%) (LIC), −2.1% (95% CI: −3.3 to −0.7%) (LMIC) and −2.7% (95% CI: −4.5 to −0.7%) (UMIC). The slope coefficients from the segmented regression model show that there was an acceleration in the rate of decrease during the period 2006 to 2009 in LIC, LMIC and UMIC, but no further significant change in the rate of decrease from 2010 to 2013 (Table 3B, Table S2B).

**Table 3:** Decline in percentage of children starting cART with severe immunodeficiency by calendar period, reflected by (A) average change in percentage per year within each WHO guideline period and (B) rate of decrease presented as estimated slope coefficient from the segmented regression analysis. This analysis was based on 52,153 patients after imputation of missing values

	Low‐income	Lower middle‐income	Upper middle‐income	High‐income
A. Average change of % of children starting with severe immunodeficiency per year within the period (95% CI)				
2002 to 2005	1.6% (−2.1% to 5.7%)	**6.6% (2.3% to 11.3%)**	2.2% (−0.7% to 5.1%)	−1.6% (−4.8% to 1.5%)
2006 to 2009	−**3.0% (**−**4.1% to** −**1.9%)**	−**2.1% (**−**3.3% to** −**0.7%)**	−**2.7% (**−**4.5% to** −**0.7%)**	−1.2% (−3.7% to 1.3%)
2010 to 2013	−**2.1% (**−**3.4% to** −**0.8%)**	−**3.8% (**−**5.4% to** −**2.1%)**	−**2.7% (**−**4.9% to** −**0.3%)**	−**4.3% (**−**5.6% to** −**1.3%)**
B. Estimated slope coefficient on the logit scale for the reference period (RP) and contrasts (95% CI)				
2006 to 2009 (RP)	−**0.122 (**−**0.171 to** −**0.076)**	−**0.088 (**−**0.147 to** −**0.030)**	−**0.108 (**−**0.187 to** −**0.030)**	−0.059 (−0.182 to 0.064)
2002 to 2005 minus RP	**0.190 (0.014 to 0.368)**	**0.373 (0.183 to 0.569)**	**0.198 (0.058 to 0.340)**	−0.012 (−0.215 to 0.199)
2010 to 2013 minus RP	0.039 (−0.026 to 0.103)	−0.064 (−0.138 to 0.011)	−0.001 (−0.107 to 0.105)	−0.267 (−0.584 to 0.031)

*Notes:* In (A), negative values indicate reductions and positive values increases in the percentage of children starting cART with severe immunodeficiency; values can be considered significantly different from zero if the corresponding CI does not include zero (shown in bold). In (B), negative slopes for the reference period (2006 to 2009) indicate an accelerated rate of decrease, and slopes in other periods are compared with the reference period. Positive values indicate a slower rate of decrease and negative values a faster rate of decrease than the reference period. Slopes are significantly different from the reference period if the corresponding CI does not contain zero (shown in bold). cART, combination antiretroviral therapy; WHO, World Health Organization.

## 4 Discussion

In our study of about 52,000 children from 32 countries, we found that since the 2006 and 2010 WHO Guidelines addressing paediatric cART eligibility, there have been annual reductions in the proportion of children starting cART with severe immunodeficiency in almost all country income groups. By 2013, less than half of children had severe immunodeficiency when starting cART in all country income groups. Nevertheless, considerable global inequity in advanced HIV disease at cART initiation remains; in 2013, >40% of children in low‐ and middle‐income countries still started cART with severe immunodeficiency compared to <20% in HIC.

### 4.1 Relationship with WHO Treatment Guidelines

The goal of increasing paediatric cART coverage is one of the key reasons for the WHO 2015 recommendation of immediate cART irrespective of CD4 for all children 4. It is therefore encouraging that previous guideline revisions that expanded paediatric cART eligibility have been temporally associated with reduction in the proportion of children with advanced HIV disease at cART initiation. Nevertheless, except in HIC, more than 40% of children continued to start cART with severe immunodeficiency in 2013. This supports expanding immediate cART to all children, and indicates that WHO guideline changes alone are insufficient to achieve optimal treatment coverage. Ongoing and proactive engagement by WHO with ministries of health should focus on implementing paediatric testing and treatment guidelines.

Expansion of cART eligibility needs to be accompanied by efforts to ensure sufficient and sustainable access to HIV commodities as well as strengthened health systems, including trained health workers and infrastructure, which requires political will and adequate funding 33. Inequities in the proportions of children starting cART with severe immunodeficiency were not only seen between different country income groups, but also among countries in the same income group. For example, in LICs, the proportion of children starting cART with severe immunodeficiency ranged across countries from 33% to 69%. This may be partly due to the particular programmes in these countries that contributed to this analysis, and the years in which those programmes initiated the majority of children on cART. It is also possible that different levels of donor and national government funding and political will contributed to these differences.

It is somewhat surprising that there were no clear decreases in median age at cART start across all country income groups since eligibility for immediate cART irrespective of CD4 values applied initially to the youngest children (<2 years) and only expanded to those <5 years in 2013 (Figure S4) 2, 3. However, interpreting trends in age at cART initiation is complex 21. The WHO guideline shifts towards universal treatment eligibility for progressively older groups of children have been accompanied by recommendations of more effective PMTCT, namely universal lifelong cART for all pregnant and breastfeeding women (so‐called “Option B+”). By 2013, the population of two‐ to five‐year olds eligible for immediate treatment would likely have decreased, both due to fewer new infections as well as some children already having started cART at less than two years of age following the implementation of 2010 guidelines. The median age at cART initiation thus depends on the effectiveness of PMTCT programmes in preventing new infant infections, capacity for EID and early cART, as well as the backlog of older children not yet on therapy 34, 35. There was a decrease in age at cART initiation in LIC, suggesting that the WHO 2010 guideline recommendation of universal cART for all children <2 years may have had the greatest impact in the poorest countries, where EID capacity increased substantially in this period 36, 37, 38, 39. Nevertheless, there was little temporal improvement in the proportion of children <1 year with severe immunosuppression, indicating that further improvements in EID and early cART access are urgently needed.

While there was an increase in overall age at cART initiation in UMIC, this may partly be due to effectiveness of PMTCT programmes preventing new infections. These countries nevertheless experienced the steepest decline in the proportion of infants with severe immunosuppression, indicating substantial progress in achieving cART initiation before the onset of advanced disease in infants. The overall increase in median age in both UMIC and HIC is likely due to reductions in new infant infections due to effective PMTCT, with a relative increase in the proportion of long‐term survivors initiating cART. Indeed, in Southern Africa, we have shown an increase in the proportions of children <1 year and >10 years old initiating cART 40. In Europe, many older patients presenting for testing and care may be immigrants, predominantly from sub‐Saharan Africa 41, 42, 43.

### 4.2 From improving paediatric cART access to improving paediatric HIV outcomes

CD4 at cART initiation is a useful indicator of responsiveness to WHO treatment initiation guidelines and indicates the extent to which we are successfully preventing advanced HIV disease through early cART initiation 44. Early cART initiation and retention are particularly critical in infants, as disease progression without cART is rapid and associated with high mortality. However, increasing CD4 at treatment start will only result in improved paediatric outcomes if those children are retained and adherent to effective treatment. There is a much‐needed emphasis on children in the UNAIDS 90:90:90 goals which focus not just on diagnosis and treatment access, but also aim for 90% retention on cART, with 90% viral suppression 44. Indeed, the WHO 2015 treatment guidelines highlight as a research gap the effect of early cART on retention and adherence 4. The IeDEA collaboration has previously found loss‐to‐follow‐up rates in children by 18 months after cART initiation ranging from 4.1% in Asia to 21.8% in West Africa 45. A recent analysis as part of an IeDEA‐WHO Collaboration found that children <2 years of age and youth aged 15 to 24 years were least likely to be retained in care 46. Expanding paediatric cART therefore requires strategies not only to improve diagnosis and cART initiation, but also to optimize retention and support adherence across the paediatric and adolescent age spectrum. As more children are initiated on treatment at younger ages, better access to viral load monitoring to assess treatment effectiveness as well as paediatric‐friendly second‐ and third‐line drugs will become more important.

### 4.3 Strengths and limitations

This analysis included a large number of children from many countries and all income group settings. It is one of the first analyses with substantial numbers of children that initiated cART after the WHO 2010 guidelines, allowing enough time to examine their effects. The inclusion of European data where, at least in some countries, there were sufficient numbers of children initiating cART to include data up to 2012, allows for comparison between all income settings after the 2006 and 2010 guideline revisions. A key limitation of our study is that we looked at the impact of WHO guidelines and not individual country guidelines on changes in CD4 at cART initiation. Although in some cases country guidelines pre‐date WHO guidelines, they more frequently lag behind WHO guidelines, which may explain the lack of a clearer effect of WHO guidelines on CD4 at cART start. Furthermore, in some country income groups, most data were from only one or two countries. Similarly, for some countries, data may have originated from a small number of programmes or from particular years. This may partly explain the diversity in findings within the same country income group, and our results may not be generalizable to the whole country or income group. However, to reflect reality as best as possible, we used a weighted analysis approach, with more weight assigned not only to more precise estimates obtained from countries contributing many observations to our data set but also dependent on the UNAIDS estimated number of children starting cART in a given country and year 31, so that countries with many children starting cART were adequately represented in our analysis. It is also possible that the facilities included in our analysis reflect better access to paediatric cART than occurs in the whole country or income group. However, almost all sites from LMICs in our cohorts are routine care facilities rather than dedicated research cohorts, and followed the relevant national cART guidelines. The routine nature of the data is reflected in the substantial proportions of missing CD4 data at cART initiation, with almost one third of patients missing a CD4 measurement in LIC. We conducted multiple imputation to minimize bias due to missing data. Results including the imputed values were very similar to those of complete case analyses. However, it is possible that there is residual bias. For example if children with poorer health were less likely to have CD4 measurements performed, this would violate the assumption of values “missing at random” 30 and lead to an underestimation of the proportion of children starting cART with severe immunodeficiency.

## 5 Conclusions

The results of this study represent a milestone in efforts to increase paediatric cART access worldwide. We are almost halfway there. In 2012/2013, for the first time in all country income groups, approximately half of children initiating cART did not have severe immunodeficiency. This is at least partly attributable to WHO guidelines changes that expanded paediatric cART eligibility. Nevertheless, the persistence of disparities in cART access across income group settings are also reflected in our data showing that in 2013 there were still >40% of children starting cART with severe immunosuppression in LMICs. The WHO 2016 guidelines provide an opportunity to expand paediatric cART access to infants, children and adolescents and encourage monitoring of the effect of the guidelines not only on immunological status at cART initiation, but also on retention and treatment effectiveness to comprehensively evaluate the effectiveness of paediatric cART programmes.

## Competing interests

There are no competing interests to declare.

## Authors’ contributions

ME, MAD, KWK, RV, CY, VL, AHS, AJ, AE, MY, GRS III, K Patel, JP and RVD designed the research study. K Panayidou and NA analysed the data. K Panayidou, ME and MAD wrote the first draft of the manuscript. All authors contributed to revising the manuscript and read and approved the final version.

## Cohort Collaboration Steering Groups:

### IeDEA Asia‐Pacific

TREAT Asia Pediatric HIV Observational Database – Steering Committee: PS Ly*, and V Khol, National Centre for HIV/AIDS, Dermatology and STDs, Phnom Penh, Cambodia; J Tucker, New Hope for Cambodian Children, Phnom Penh, Cambodia; N Kumarasamy*, S Saghayam, and E Chandrasekaran, YRGCARE Medical Centre, CART CRS, Chennai, India; DK Wati*, D Vedaswari, and IY Malino, Sanglah Hospital, Udayana University, Bali, Indonesia; N Kurniati*, and D Muktiarti, Cipto Mangunkusumo – Faculty of Medicine Universitas Indonesia, Jakarta, Indonesia; SM Fong*, M Lim, and F Daut, Hospital Likas, Kota Kinabalu, Malaysia; NK Nik Yusoff*‡, and P Mohamad, Hospital Raja Perempuan Zainab II, Kelantan, Malaysia; TJ Mohamed* and MR Drawis, Pediatric Institute, Hospital Kuala Lumpur, Kuala Lumpur, Malaysia; R Nallusamy*, and KC Chan, Penang Hospital, Penang, Malaysia; T Sudjaritruk*, V Sirisanthana, L Aurpibul, and P Oberdorfer, Department of Pediatrics, Faculty of Medicine, Chiang Mai University and Research Institute for Health Sciences, Chiang Mai, Thailand; R Hansudewechakul*, S Denjanta, S Watanaporn, and A Kongphonoi, Chiangrai Prachanukroh Hospital, Chiang Rai, Thailand; P Lumbiganon*†, P Kosalaraksa, P Tharnprisan, and T Udomphanit, Division of Infectious Diseases, Department of Pediatrics, Faculty of Medicine, Khon Kaen University, Khon Kaen, Thailand; G Jourdain, PHPT‐IRD UMI 174 (Institut de recherche pour le développement and Chiang Mai University), Chiang Mai, Thailand; T Puthanakit*, S Anugulruengkitt, and C Phadungphon, HIV‐NAT, The Thai Red Cross AIDS Research Centre, Bangkok, Thailand; K Chokephaibulkit*, K Lapphra, W Phongsamart, and S Sricharoenchai, Department of Pediatrics, Faculty of Medicine Siriraj Hospital, Mahidol University, Bangkok, Thailand; KH Truong*, QT Du, and CH Nguyen, Children's Hospital 1, Ho Chi Minh City, Vietnam; VC Do*, TM Ha, and VT An Children's Hospital 2, Ho Chi Minh City, Vietnam; LV Nguyen*, DTK Khu, AN Pham, and LT Nguyen, National Hospital of Pediatrics, Hanoi, Vietnam; ON Le, Worldwide Orphans Foundation, Ho Chi Minh City, Vietnam; AH Sohn*, JL Ross, and C Sethaputra, TREAT Asia/amfAR – The Foundation for AIDS Research, Bangkok, Thailand; MG Law* and A Kariminia, The Kirby Institute, UNSW Australia, Sydney, Australia; (*Steering Committee members; †Current Steering Committee Chair; ‡co‐Chair).

### IeDEA Caribbean, Central, and South America (CCASAnet)

Fundación Huésped, Argentina: Pedro Cahn, Carina Cesar, Valeria Fink, Omar Sued, Emanuel Dell'Isola, Hector Perez, Jose Valiente, Cleyton Yamamoto; Instituto Nacional de Infectologia‐Fiocruz, Brazil: Beatriz Grinsztejn, Valdilea Veloso, Paula Luz, Raquel de Boni, Sandra Cardoso Wagner, Ruth Friedman, Ronaldo Moreira; Universidade Federal de Minas Gerais, Brazil: Jorge Pinto, Flavia Ferreira, Marcelle Maia; Universidade Federal de São Paulo, Brazil: Regina Célia de Menezes Succi, Daisy Maria Machado, Aida de Fátima Barbosa Gouvêa; Fundación Arriarán, Chile: Marcelo Wolff, Claudia Cortes, Maria Fernanda Rodriguez, Gladys Allendes; Les Centres GHESKIO, Haiti: Jean William Pape, Vanessa Rouzier, Adias Marcelin, Christian Perodin; Hospital Escuela Universitario, Honduras: Marco Tulio Luque; Instituto Hondureño de Seguridad Social, Honduras: Denis Padgett; Instituto Nacional de Ciencias Médicas y Nutrición Salvador Zubirán, Mexico: Juan Sierra Madero, Brenda Crabtree Ramirez, Paco Belaunzaran, Yanink Caro Vega; Instituto de Medicina Tropical Alexander von Humboldt, Peru: Eduardo Gotuzzo, Fernando Mejia, Gabriela Carriquiry; Vanderbilt University Medical Center, USA: Catherine C McGowan, Bryan E Shepherd, Timothy Sterling, Karu Jayathilake, Anna K Person, Peter F Rebeiro, Mark Giganti, Jessica Castilho, Stephany N Duda, Fernanda Maruri, Hilary Vansell.

### Central Africa IeDEA

Site investigators and cohorts: Nimbona Pélagie, ANSS, Burundi; Patrick Gateretse, Jeanine Munezero, Valentin Nitereka, Théodore Niyongabo, Christelle Twizere, Centre National de Reference en Matiere de VIH/SIDA, Burundi; Hélène Bukuru, Thierry Nahimana, CHUK, Burundi; Jérémie Biziragusenyuka, Risase Scholastique Manyundo, HPRC, Burundi; Kien Atsu, Tabeyang Mbuh, Bamenda Hospital, Cameroon; Rogers Ajeh, Mark Benwi, Anastase Dzudie, Akindeh Mbuh, Marc Lionel Ngamani, Victorine Nkome, CRENC & Douala General Hospital, Cameroon; Djenabou Amadou, Eric Ngassam, Eric Walter Pefura Yone, Jamot Hospital, Cameroon; Alice Ndelle Ewanoge, Norbert Fuhngwa, Chris Moki, Limbe Regional Hospital, Cameroon; Catherine Akele, Faustin Kitetele, Patricia Lelo, Martine Tabala, Kalembelembe Pediatric Hospital, Democratic Republic of Congo; Emile Wemakoy Okitolonda, Landry Wenzi, Kinshasa School of Public Health, Democratic Republic of Congo; Merlin Diafouka, Martin Herbas Ekat, Dominique Mahambou Nsonde, CTA Brazzaville, Republic of Congo; Adolphe Mafou, CTA Pointe‐Noire, Republic of Congo; Fidele Ntarambirwa, Bethsaida Hospital, Rwanda; Yvonne Tuyishimire, Busanza Health Center, Rwanda; Theogene Hakizimana, Gahanga Health Center, Rwanda; Josephine Ayinkamiye, Gikondo Health Center, Rwanda; Sandrine Mukantwali, Kabuga Health Center, Rwanda; Henriette Kayitesi, Olive Uwamahoro, Kicukiro Health Center, Rwanda; Viateur Habumuremyi, Masaka Health Center, Rwanda; Joyce Mukamana, Nyarugunga Health Center, Rwanda; Jean Claude Dusingize, Gallican Kubwimana, Pacifique Mugenzi, Benjamin Muhoza, Athanase Munyaneza, Emmanuel Ndahiro, Diane Nyiransabimana, Jean d'Amour Sinayobye, Vincent Sugira, Rwanda Military Hospital, Rwanda; Chantal Benekigeri, Gilbert Mbaraga, WE‐ACTx Health Center, Rwanda. Coordinating and Data Centers: Adebola Adedimeji, Kathryn Anastos, Madeline Dilorenzo, Lynn Murchison, Jonathan Ross, Albert Einstein College of Medicine, USA; Diane Addison, Meg Baker, Ellen Brazier, Heidi Jones, Elizabeth Kelvin, Sarah Kulkarni, Denis Nash, Olga Tymejczyk, City University of New York (CUNY), School of Public Health, USA; Batya Elul, Columbia University, USA; Xiatao Cai, Don Hoover, Hae‐Young Kim, Chunshan Li, Qiuhu Shi, Data Solutions, USA; Kathryn Lancaster, Marcel Yotebieng, Ohio State University, USA; Mark Kuniholm, University at Albany, State University of New York, USA; Andrew Edmonds, Angela Parcesepe, University of North Carolina at Chapel Hill, USA; Stephany Duda; Vanderbilt University School of Medicine, USA; April Kimmel, Virginia Commonwealth University School of Medicine, USA; Margaret McNairy, Weill Cornell Medical Center.

### East Africa IeDEA

Lameck Diero, Samuel Ayaya, AMPATH Plus, MOI University Eldoret, Kenya; Elizabeth Bukusi, Kenya Medical Research Institute (KEMRI), Kisumu, Kenya; John Ssali, Masaka Regional Referral Hospital, Masaka, Uganda; Fred Nalugoda, Rakai Health Sciences Program, Kalisizo, Uganda; G.R. Somi, National AIDS Control Program (NACP) Dar es Salaam, Tanzania; Rita Elias Lyamuya, Morogoro Regional Hospital, Morogoro, Tanzania; Kapella Ngonyani, Tumbi Regional Hospital, Pwani, Tanzania; and Emanuel Lugina, Ocean Road Cancer Institute, Dar es Salaam, Tanzania; Mark Urassa, Denna Michael, National Institute for Medical Research (NIMR) Kisesa HDSS, Mwanza, Tanzania.

### West Africa IeDEA

Site investigators and cohorts: Adult cohorts: Marcel Djimon Zannou, CNHU, Cotonou, Benin; Armel Poda, CHU Souro Sanou, Bobo Dioulasso, Burkina Faso; Fred Stephen Sarfo, Komfo Anokeye Teaching Hospital, Kumasi, Ghana; Eugene Messou, ACONDA CePReF, Abidjan, Ivory Coast; Henri Chenal, CIRBA, Abidjan, Ivory Coast; Kla Albert Minga, CNTS, Abidjan, Ivory Coast; Emmanuel Bissagnene, & Aristophane Tanon, CHU Treichville, Ivory Coast; Moussa Seydi, CHU de Fann, Dakar, Senegal; Akessiwe Akouda Patassi, CHU Sylvanus Olympio, Lomé, Togo.Pediatric cohorts: Sikiratou Adouni Koumakpai‐Adeothy, CNHU, Cotonou, Benin; Lorna Awo Renner, Korle Bu Hospital, Accra, Ghana; Sylvie Marie N'Gbeche, ACONDA CePReF, Abidjan, Ivory Coast; Clarisse Amani Bosse, ACONDA_MTCT+, Abidjan, Ivory Coast; Kouadio Kouakou, CIRBA, Abidjan, Ivory Coast; Madeleine Amorissani Folquet, CHU de Cocody, Abidjan, Ivory Coast; François Tanoh Eboua, CHU de Yopougon, Abidjan, Ivory Coast; Fatoumata Dicko Traore, Mariam Sylla, Hopital Gabriel Toure, Bamako, Mali; Elom Takassi, CHU Sylvanus Olympio, Lomé,Togo Coordinating & data centers: François Dabis, Elise Arrive, Eric Balestre, Renaud Becquet, Charlotte Bernard, Shino Chassagne Arikawa, Alexandra Doring, Antoine Jaquet, Karen Malateste, Elodie Rabourdin, Thierry Tiendrebeogo : ADERA, Isped & Inserm U1219, Bordeaux, France. Sophie Desmonde, Julie Jesson, Valeriane Leroy : Inserm U1027, Toulouse, France. Didier Koumavi Ekouevi, Jean‐Claude Azani, Patrick Coffié, Guy Gnepa, Christian Gerard Kaugbouh Kouadio, Boris Tchounga : PACCI, CHU Treichville, Abidjan, Ivory Coast

### IeDEA Southern Africa

Gary Maartens, Aid for AIDS, South Africa; Michael Vinikoor, Centre for Infectious Disease Research in Zambia (CIDRZ), Zambia; Monique von Lettow, Dignitas, Malawi; Robin Wood, Gugulethu ART Programme, South Africa; Shobna Sawry, Harriet Shezi Children's Clinic, South Africa; Frank Tanser, Africa Health Research Institute (Hlabisa), South Africa & School of Nursing and Public Health, University of KwaZulu‐Natal, Durban, South Africa; Andrew Boulle, Khayelitsha ART Programme, South Africa; Geoffrey Fatti, Kheth'Impilo, South Africa; Sam Phiri, Lighthouse Clinic, Malawi; Cleophas Chimbetete, Newlands Clinic, Zimbabwe; Karl‐Günther Technau, Rahima Moosa Mother and Child Hospital, South Africa; Brian Eley, Red Cross Children's Hospital, South Africa; Josephine Muhairwe, SolidarMed Lesotho; Anna Jores, SolidarMed Mozambique; Kamelia Kamenova, SolidarMed Zimbabwe, Matthew P Fox, Themba Lethu Clinic, South Africa; Hans Prozesky, Tygerberg Academic Hospital, South Africa.

## COHERE

Steering Committee – Contributing Cohorts: Ali Judd (AALPHI), Robert Zangerle (AHIVCOS),Giota Touloumi (AMACS), Josiane Warszawski (ANRS CO1 EPF/ANRS CO11 OBSERVATOIRE EPF), Laurence Meyer (ANRS CO2 SEROCO), François Dabis (ANRS CO3 AQUITAINE), Murielle Mary Krause (ANRS CO4 FHDH), Jade Ghosn (ANRS CO6 PRIMO), Catherine Leport (ANRS CO8 COPILOTE), Linda Wittkop (ANRS CO13 HEPAVIH), Peter Reiss (ATHENA), Ferdinand Wit (ATHENA), Maria Prins (CASCADE), Heiner Bucher (CASCADE), Diana Gibb (CHIPS), Gerd Fätkenheuer (Cologne‐Bonn), Julia Del Amo (CoRIS), Niels Obel (Danish HIV Cohort), Claire Thorne (ECS), Amanda Mocroft (EuroSIDA), Ole Kirk (EuroSIDA), Christoph Stephan (Frankfurt), Santiago Pérez‐Hoyos (GEMES‐Haemo), Osamah Hamouda (German ClinSurv), Barbara Bartmeyer (German ClinSurv), Nikoloz Chkhartishvili (Georgian National HIV/AIDS), Antoni Noguera‐Julian (CORISPE‐cat), Andrea Antinori (ICC), Antonella d'Arminio Monforte (ICONA), Norbert Brockmeyer (KOMPNET), Luis Prieto (Madrid PMTCT Cohort), Pablo Rojo Conejo (CORISPES‐Madrid), Antoni Soriano‐Arandes (NENEXP), Manuel Battegay (SHCS), Roger Kouyos (SHCS), Cristina Mussini (Modena Cohort), Pat Tookey (NSHPC), Jordi Casabona (PISCIS), Jose M. Miro (PISCIS), Antonella Castagna (San Raffaele), Deborah_Konopnick (St. Pierre Cohort), Tessa Goetghebuer (St Pierre Paediatric Cohort), Anders Sönnerborg (Swedish InfCare), Carlo Torti (The Italian Master Cohort), Caroline Sabin (UK CHIC), Ramon Teira (VACH), Myriam Garrido (VACH). David Haerry (European AIDS Treatment Group) Executive Committee: Stéphane de Wit (Chair, St. Pierre University Hospital), Jose M. Miro (PISCIS), Dominique Costagliola (FHDH), Antonella d'Arminio‐Monforte (ICONA), Antonella Castagna (San Raffaele), Julia del Amo (CoRIS), Amanda Mocroft (EuroSida), Dorthe Raben (Head, Copenhagen Regional Coordinating Centre), Geneviève Chêne (Head, Bordeaux Regional Coordinating Centre). Paediatric Cohort Representatives: Ali Judd, Pablo Rojo Conejo. Regional Coordinating Centres: Bordeaux RCC: Diana Barger, Christine Schwimmer, Monique Termote, Linda Wittkop; Copenhagen RCC: Maria Campbell, Casper M. Frederiksen, Nina Friis‐Møller, Jesper Kjaer, Dorthe Raben, Rikke Salbøl Brandt. Project Leads and Statisticians: Juan Berenguer, Julia Bohlius, Vincent Bouteloup, Heiner Bucher, Alessandro Cozzi‐Lepri, François Dabis, Antonella d'Arminio Monforte, Mary‐Ann Davies, Julia del Amo, Maria Dorrucci, David Dunn, Matthias Egger, Hansjakob Furrer, Marguerite Guiguet, Sophie Grabar, Ali Judd, Ole Kirk, Olivier Lambotte, Valériane Leroy, Sara Lodi, Sophie Matheron, Laurence Meyer, Jose Mª Miró, Amanda Mocroft, Susana Monge, Fumiyo Nakagawa, Roger Paredes, Andrew Phillips, Massimo Puoti, Eliane Rohner, Michael Schomaker, Colette Smit, Jonathan Sterne, Rodolphe Thiebaut, Claire Thorne, Carlo Torti, Marc van der Valk, Linda Wittkop.

## PHACS/IMPAACT

Steering committee: Mark J. Abzug, Rohan Hazra, Barbara Heckman, Ellen O'gara, James Oleske, Kunjal Patel, George R. Seage III, Russell B. Van Dyke, Paige L. Williams, Suzanne Siminski.

## Supporting information


**Appendix S1.** Supplementary material.
**Table S1.** Comparison of patients starting cART with and without CD4 cell count. Analysis of 44,480 patients included in multiple imputation and regression analyses
**Table S2.** Decline in percentage of children starting cART with severe immunodeficiency by calendar period, reflected by (A) average change in percentage per year within each WHO guideline period and (B) rate of decrease presented as estimated slope coefficient from the segmented regression analysis. This analysis was based on complete cases (34,363 children)
**Figure S1.** Flow chart of children included and excluded from analyses.
**Figure S2.** Severe immunodeficiency at the start of cART by age (rows), sex (columns) and country income groups (colours). Results from generalized additive mixed effects models based on 34,363 children with complete data. 95% CIs are shown as shaded areas.
**Figure S3.** Median CD4 cell count in children aged 5 years or older and median CD4% in children below 5 years of age at the start of cART by age (rows), sex (columns) and income group (colours). Results from generalized additive mixed effects models based on 34,363 children with complete data. 95% CIs are shown as shaded areas.
**Figure S4.** Median age in years at start of cART by income group. Analysis based on 52,153 patients.

## Appendix 1 Composition of the Writing Group

**IeDEA Southern Africa:** Geoffrey Fatti (Orcid ID: 0000‐0002‐6467‐662X)^3^, Michael Vinikoor^4,5,6^, Shobna Sawry (Orcid ID: 0000‐0002‐3845‐4863)^7^, Jochen Ehmer (Orcid ID: 0000‐0001‐5942‐3743)^8^, Brian Eley (Orcid ID: 0000‐0003‐0811‐0098)^9^, Sam Phiri^10^, Karl‐Günter Technau (Orcid ID: 0000‐0001‐7367‐7512)^11^, Cleophas Chimbetete^12^, Helena Rabie^13^, Andrew Boulle^2,14^, Frank Tanser (Orcid ID: 0000‐0001‐9797‐0000)^15^, Robin Wood^16^

**IeDEA East Africa:** Kara Wools‐Kaloustian (Orcid ID: 0000‐0002‐3277‐4452)^17^, Rachel Vreeman (Orcid ID: 0000‐0001‐5460‐8204)^18^, Patrick Oyaro^19^, Samuel Ayaya^20^, Gertrude Nakigozi^21^, Beverley Musick^22^, Constantin Yiannoutsos^22^

**IeDEA West Africa:** Madeleine Amorissani‐Folquet^23^, Elom Takassi^24^, Mariam Sylla^25^, Lorna Renner^26^, Karen Malateste^27^, Sophie Desmonde^28^, Valériane Leroy (Orcid ID: 0000‐0003‐3542‐8616)^28^

**IeDEA Asia‐Pacific:** Nia Kurniati^29^, Rawiwan Hansudewechakul^30^, Lam Van Nguyen^31^, Penh Sun Ly^32^, Khanh Huu Truong^33^, Azar Kariminia^34^, Annette H. Sohn (Orcid ID: 0000‐0002‐0209‐3285)^35^

**IeDEA Central Africa:** Andrew Edmonds^36^, Habakkuk Azinyui Yumo (Orcid ID: 0000‐0001‐5192‐0964)^37^, Jean Claude Dusingize^38^, Marcel Yotebieng (Orcid ID: 0000‐0003‐2110‐2631)^39^

**COHERE:** Ali Judd (Orcid ID: 0000‐0003‐3176‐5295)^40^, Pablo Rojo (Orcid ID: 0000‐0002‐0197‐5462)^41^, Colette Smit^42^, Sophie Grabar (Orcid ID: 0000‐0002‐4816‐4261)^43,44^, Josiane Warszwarski^45^, Genevieve Chene^46^, Dorthe Raban^47^

**IMPAACT 219C and PHACS:** Kunjal Patel^48^, George R. Seage III^48^, Russell B. Van Dyke (Orcid ID: 0000‐0003‐3265‐3826)^49^,James Oleske^50^, Paige L. Williams^48^, Mark J. Abzug^51^

**CCASAnet:** Regina Succi (Orcid ID: 0000‐0003‐4522‐6292)^52^, Daisy M. Machado (Orcid ID: 0000‐0003‐1993‐6442)^52^, Jorge Pinto^53^, Vanessa Rouzier^54^, Marco Luque^55^, Fernando Mejia^56^

**Affiliations:** ^1^Institute of Social and Preventive Medicine, University of Bern, Switzerland; ^2^Centre for Infectious Disease Epidemiology and Research, School of Public Health and Family Medicine, University of Cape Town, South Africa; ^3^Kheth'Impilo, Cape Town, and Division of Epidemiology and Biostatistics, Department of Global Health, Faculty of Medicine and Health Sciences, Stellenbosch University, South Africa; ^4^Department of Medicine, University of Alabama at Birmingham, Birmingham, AL, USA; ^5^Centre for Infectious Disease Research in Zambia, Lusaka, Zambia; ^6^Department of Medicine, University of Zambia, Lusaka, Zambia; ^7^Harriet Shezi Children's Clinic, Soweto, and Wits Reproductive Health and HIV Institute, University of Witwatersrand, Johannesburg, South Africa; ^8^SolidarMed, Swiss Organization for Health in Africa, Lucerne, Switzerland; ^9^Red Cross War Memorial Children's Hospital and Department of Paediatrics and Child Health, University of Cape Town, South Africa; ^10^Lighthouse Trust Clinic, Lilongwe, Malawi; ^11^Empliweni Services and Research Unit, Rahima Moosa Hospital and University of the Witwatersrand, South Africa; ^12^Newlands Clinic, Harare, Zimbabwe; ^13^Tygerberg Academic Hospital, University of Stellenbosch, South Africa; ^14^Médecins Sans Frontières and the Khayelitsha ART Program, Khayelitsha, South Africa; ^15^Africa Health Research Institute, School of Nursing and Public Health, University of KwaZulu‐Natal, Durban, and Hlabisa HIV Programme, South Africa; ^16^Gugulethu HIV Program and Desmond Tutu HIV Centre, University of Cape Town, South Africa; ^17^Indiana University School of Medicine, Department of Medicine, Indianapolis, IN, USA; ^18^Indiana University School of Medicine, Department of Pediatrics, Indianapolis, IN, USA; ^19^Family AIDS Care and Education Services, Kisumu, Kenya; ^20^MOI University, Department of Child Health and Pediatrics, Eldoret, Kenya; ^21^Rakai Health Sciences Program, Kalisizo, Uganda; ^22^Indiana University, R.M. Fairbanks School of Public Health and Indiana School of Medicine, Departments of Biostatistics, Indianapolis, IN; ^23^CHU de Cocody, Abidjan, Côte d'Ivoire; ^24^CHU Sylvanus Olympio, Lomé, Togo; ^25^CHU Gabriel Touré, Bamako, Mali; ^26^KorleBu Hospital, Accra, Ghana, ^27^Inserm, UMR1219, Université de Bordeaux, Bordeaux, France, ^28^Inserm, UMR1027, Université de Toulouse 3, Toulouse, France; ^29^Cipto Mangunkusumo General Hospital, Jakarta, Indonesia; ^30^Chiangrai Prachanukroh Hospital, Chiang Rai, Thailand; ^31^National Hospital of Pediatrics, Hanoi, Vietnam; ^32^National Centre for HIV/AIDS, Dermatology and STDs, Phnom Penh, Cambodia; ^33^Children's Hospital 1, Ho Chi Minh City, Vietnam; ^34^The Kirby Institute, UNSW, Sydney, Australia; ^35^TREAT Asia/amfAR – The Foundation for AIDS Research, Bangkok, Thailand; ^36^Department of Epidemiology, The University of North Carolina at Chapel Hill, Chapel Hill, NC, USA; ^37^Research for Development (R4D) International, Yaoundé, Cameroon; ^38^Division of Research and Clinical Education, The Rwanda Military Hospital, Kanombe, Kigali Rwanda; ^39^College of Public Health, Division of Epidemiology, The Ohio State University, Columbus, Ohio, USA; ^40^MRC Clinical Trials Unit, University College London, London, UK; ^41^Department of Pediatrics. Hospital 12 de Octubre. Universidad Complutense de Madrid, Spain; ^42^Stichting HIV Monitoring, Amsterdam, The Netherlands; ^43^Sorbonne Universités, UPMC Univ Paris 06 and INSERM, UMR_S 1136, Institut Pierre Louis d'Epidémiologie et de Santé Publique, F‐75,013, Paris, France; ^44^Université Paris Descartes et Assistance Publique‐Hôpitaux de Paris, Groupe hospitalier Cochin Hôtel‐Dieu, Paris, France; ^45^Centre de recherche en épidémiologie et santé des populations, 1018 Inserm, France; ^46^Inserm, UMR1219, Bordeaux Population Health Research Center, Univ. Bordeaux, ISPED/Bordeaux School of Public Health, Bordeaux, France; ^47^Department of Infectious Diseases, Rigshospitalet, University of Copenhagen, Copenhagen, Denmark; ^48^Department of Epidemiology, Center for Biostatistics in AIDS Research, Harvard T.H. Chan School of Public Health, Boston, MA, USA; ^49^Department of Pediatrics, Tulane University School of Medicine, New Orleans, Louisiana; ^50^Division of Pediatric Allergy, Immunology and Infectious Diseases, New Jersey Medical School at Rutgers, Newark; ^51^Department of Pediatrics (Infectious Diseases), University of Colorado School of Medicine and Children's Hospital Colorado, Aurora, CO, USA; ^52^Escola Paulista de Medicina, Universidade Federal de São Paulo, São Paulo, Brazil; ^53^Universidade Federal de Minas Gerais, Belo Horizonte, Brazil; ^54^Les Centres GHESKIO, Port‐au‐Prince, Haiti; ^55^Hospital Escuela Universitario, Tegucigalpa, Honduras; ^56^Instituto de Medicina Tropical Alexander von Humboldt, Lima, Peru.
