# Supplementary material for: Global temporal changes in the proportion of children with advanced disease at the start of combination antiretroviral therapy in an era of changing criteria for treatment initiation
Source: J Int AIDS Soc. 2018 Nov 22;21(11):e25200. doi: 10.1002/jia2.25200 (PMC6275813; doi:10.1002/jia2.25200)
Supplement: Supplementary file 1 — Appendix S1. Supplementary material. Table S1. Comparison of patients starting cART with and without CD4 cell count. Analysis of 44,480 patients included in multiple imputation and regression analyses Table S2. Decline in percentage of children starting cART with severe immunodeficiency by calendar period, reflected by (A) average change in percentage per year within each WHO guideline period and (B) rate of decrease presented as estimated slope coefficient from the segmented regression analysis. This analysis was based on complete cases (34,363 children) Figure S1. Flow chart of children included and excluded from analyses. Figure S2. Severe immunodeficiency at the start of cART by age (rows), sex (columns) and country income groups (colours). Results from generalized additive mixed effects models based on 34,363 children with complete data. 95% CIs are shown as shaded areas. Figure S3. Median CD4 cell count in children aged 5 years or older and median CD4% in children below 5 years of age at the start of cART by age (rows), sex (columns) and income group (colours). Results from generalized additive mixed effects models based on 34,363 children with complete data. 95% CIs are shown as shaded areas. Figure S4. Median age in years at start of cART by income group. Analysis based on 52,153 patients. [file JIA2-21-e25200-s001.docx]

**APPENDIX: Supplementary material**

**Technical appendix**

**Multiple imputation**

The multiple imputation was carried out using the *mice* package in R (R Core Team, Vienna, Austria)^^[[1]](#footnote-1)^^ under the assumption that observations were missing at random. We imputed the square root of CD4 count and arcsine square root of CD4% simultaneously using predictive mean matching and chained equations with the default configuration options. To allow for interaction terms in the analyses, we stratified the multiple imputation by age, sex and income groups. We included the variables country and year of starting cART. We coded the variable year of cART start and all the other variables as categorical variables. To minimize variability, we created 50 imputed datasets.

Multiple imputation of missing CD4 measurements was based on 52,153 patients. Compared to 17,790 (34%) patients who had CD4 measurement missing, the 34,363 patients with a measurement were older and less likely to be from a LIC (Table S1). The CD4 count and percentage was missing in 30% of patients in LIC, 22% in LMIC, 26% in UMIC and 11% in HIC (Table 2). CD4 counts were imputed for 6,478 (22%) of 29,157 children aged ≥5 years with no CD4 measure and for 576 (2%) of children with only CD4% available. Similarly CD4% values were imputed for 5,962 (25%) of 23,478 children aged <5 years with no CD4 measure and for 3,530 (15%) children who had a CD4 count available. Medians of complete case and imputed CD4 data were similar for most countries and only differed by more than 20 cells/µl in absolute terms for males in Mozambique, and Peru, females in France and both sexes in Spain (Table 2). The percentages of children starting cART with severe immunodeficiency based on imputed CD4 data and complete cases were generally similar; only Tanzania had a difference of more than 10% in absolute terms (Table 2).

**Computation of smoothers**

We used the R package *gamm4* in R 3.1 (R Core Team, Vienna, Austria)^^[[2]](#footnote-2)^^ to fit generalized additive mixed models. Basis representations were constructed using tensor products. Basis construction was done for year; for year and age group; for year, age group and income group as well as for year, age group, income group and sex. Collinear terms in the bases were subsequently removed. We included a random intercept for every country. More complex random effects structures – such as a random slope – did not improve the model fit. We fitted the model separately for each imputed dataset and combined predicted values and standard errors in a point wise manner. To get estimates of the median CD4 counts, median CD4% and percentage of children starting with severe immunodeficiency, we aggregated the data for each combination of year, sex, country and age group (the predictors) computing the median and the number of patients below the threshold for each combination. We used the aggregated data in all the standard and generalized additive model analyses. To account for the varying number of observations available for each combination of the predictors, we used weights in all the analyses where the aggregated data was used.

Table S1. Comparison of patients starting cART with and without CD4 cell count. Analysis of 44,480 patients included in multiple imputation and regression analyses.

| **Variable** | | **CD4 cell count (age >= 5) or CD4% (age < 5) at start of cART available** | **CD4 cell count (age >= 5) or CD4% (age < 5) at start of cART missing** |
| --- | --- | --- | --- |
|  |  | **(n=34,363)** | **(n=17,790)** |
| **Median age (years; IQR)** | | 6.8 (3.0-10.5) | 3.8 (1.6-8.2) |
| **Sex** | |  |  |
|  | Male | 17,023 (50%) | 8,935 (50%) |
|  | Female | 17,340 (50%) | 8,855 (50%) |
| **Country income level** | |  |  |
|  | Low | 12,747 (37%) | 8,525 (48%) |
|  | Lower middle | 13,541 (39%) | 5,782 (33%) |
|  | Upper middle | 5,287 (15%) | 3,051 (17%) |
|  | High | 2,788 (8%) | 432 (2%) |
| **Median year of starting cART (IQR)** | | 2009 (2007-2010) | 2009 (2007-2011) |
| IQR: interquartile range. | |  |  |

Table S2: Decline in percentage of children starting cART with severe immunodeficiency by calendar period, reflected by (A) average change in percentage per year within each WHO guideline period, and (B) rate of decrease presented as estimated slope coefficient from the segmented regression analysis. This analysis was based on complete cases (34,363 children).

|  | **Low-income** | **Lower middle-income** | **Upper middle-income** | **High-income** |
| --- | --- | --- | --- | --- |
| **Average change of % of children starting with severe immunodeficiency per year within the period (95% CI)** | | | | |
| **2002 to 2005** | 3.1% (-0.5% to 6.9%) | 3.7% (-1.1% to 8.4%) | 1.8% (-1.2% to 4.9%) | -1.4% (-5.5% to 2.0%) |
| **2006 to 2009** | **-3.3% (-4.7% to -2.0%)** | **-2.2% (-3.9% to -0.5%)** | **-2.4% (-4.6% to -0.1%)** | -1.0% (-3.8% to 1.8%) |
| **2010 to 2013** | **-3.3% (-4.6% to -1.9%)** | **-4.3% (-6.3% to -2.4%)** | **-3.4% (-5.7% to -0.8%)** | **-5.0% (-6.7% to -1.6%)** |
| **Estimated slope co-efficient on the logit scale for the reference period (RP) and contrasts (95% CI)** | | | | |
| **2006 to 2009 (RP)** | **-0.135 (-0.192 to -0.080)** | **-0.090 (-0.165 to -0.020)** | **-0.097 (-0.196 to -0.003)** | -0.049 (-0.189 to 0.090) |
| **2002 to 2005 minus RP** | **0.262 (0.125 to 0.429)** | **0.248 (0.030 to 0.461)** | **0.171 (0.020 to 0.310)** | -0.014 (-0.257 to 0.230) |
| **2010 to 2013 minus RP** | 0.001 (-0.064 to 0.062) | **-0.084 (-0.160 to -0.003)** | -0.039 (-0.137 to 0.064) | -0.320 (-0.688 to 0.000) |

NOTES:

In Panel (A), negative values indicate reductions and positive values increases in the percentage of children starting ART with severe immunodeficiency; values can be considered significantly different from zero if the corresponding CI does not include zero (shown in bold).

In Panel (B), negative slopes for the reference period (2006-2009) indicate an accelerated rate of decrease, and slopes in other periods are compared with the reference period.  Positive values indicate a slower rate of decrease and negative values a faster rate of decrease than the reference period.  Slopes are significantly different from the reference period if the corresponding CI does not contain zero (shown in bold).

Figure S1. Flow chart of children included and excluded from analyses.

1,034,781 (100%)

Data received

921,447 (89.0%)

- age at cART start >15

45,848 (4.4%)

- missing cART start date and missing age at cART start

67,486 (6.5%)

Children starting ART

54,103 (5.2%)
Included in descriptive analyses

52,153 (5.0%)
Included in imputed-data analyses

34,363 (3.3%)
Included in complete case analyses

8 (<1%)

- missing sex

13,316 (1.3%)

- not treatment naïve

59 (<1%)

- started before 1995

268 (<1%)

- cART start year before 1996 (HIC) / 2002 (LIC, LMIC, UMIC)

138 (<1%)

- last observation in last year in country before May

1544 (<1%)

- < 10 patients with non-missing CD4 measurement in a country for a particular year

0 (0.0%)

- <50 patients with non-missing CD4
  measurement / country

17,790 (1.7%)

- missing CD4 cell count measurement

Figure S2. Severe immunodeficiency at the start of cART by age (rows), sex (columns) and country income groups (colors). Results from generalized additive mixed effects models based on 34,363 children with complete data. 95% CIs are shown as shaded areas.


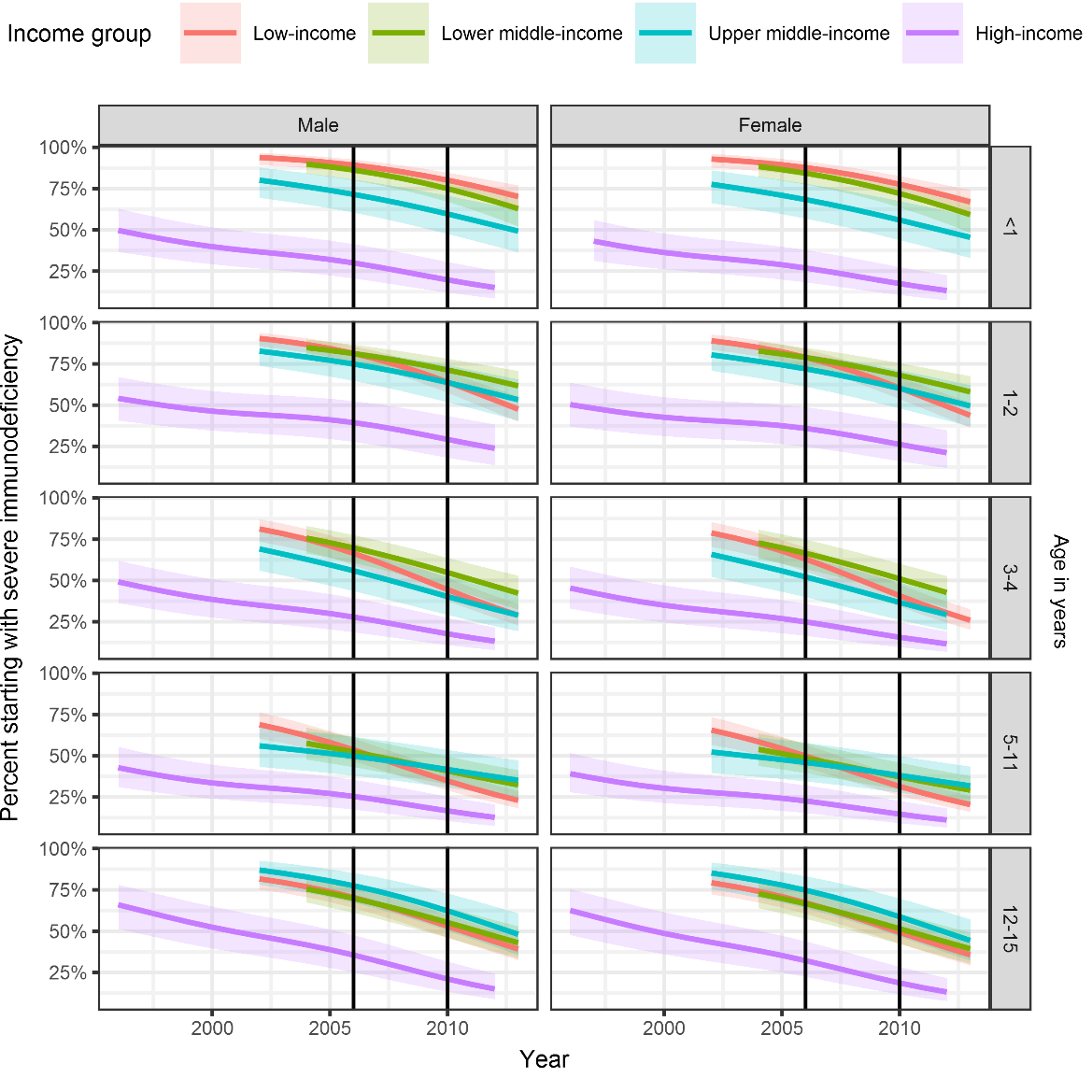


Figure S3. Median CD4 cell count in children aged 5 years or older and median CD4% in children below 5 years of age at the start of cART by age (rows), sex (columns) and income group (colors). Results from generalized additive mixed effects models based on 34,363 children with complete data. 95% CIs are shown as shaded areas.


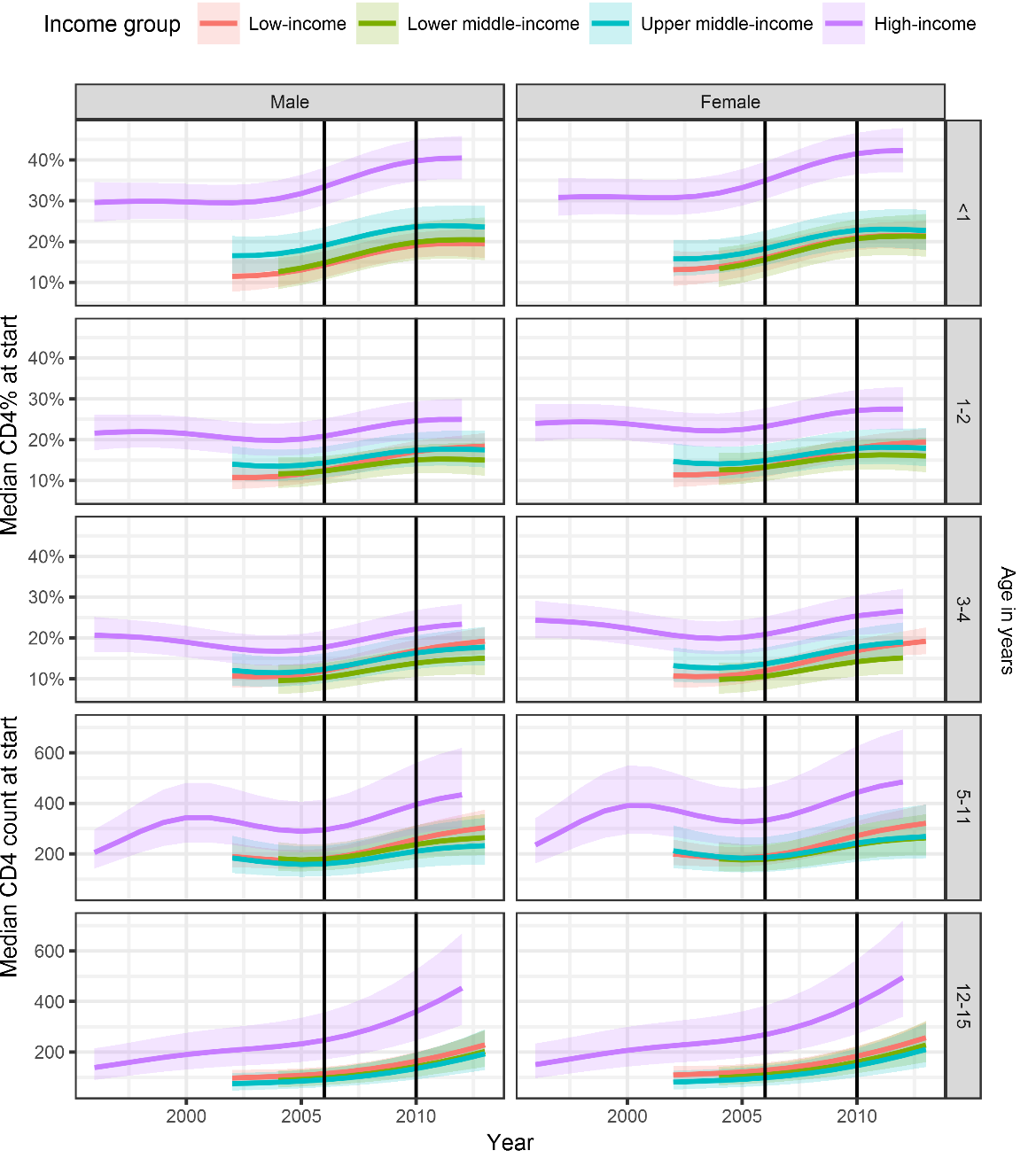


Figure S4.Median age in years at start of cART by income group. Analysis based on 52,153 patients.


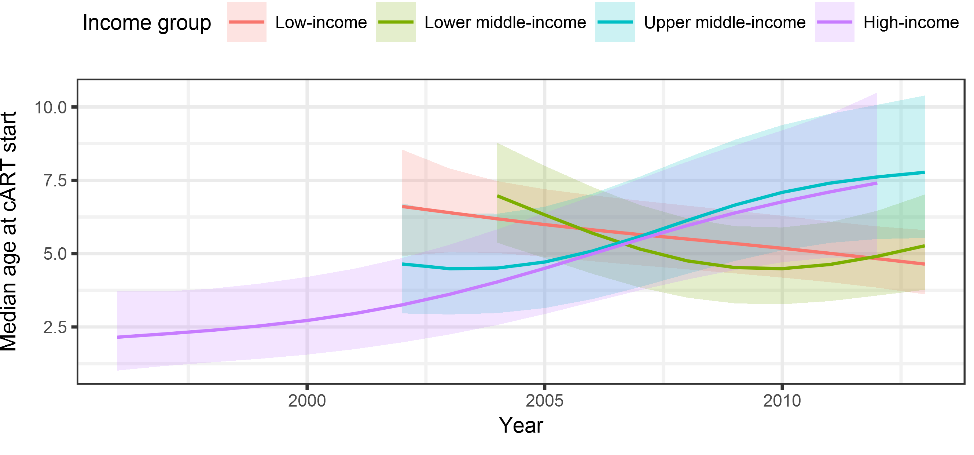


1. Stef van Buuren, Karin Groothuis-Oudshoorn (2011). mice: Multivariate Imputation by Chained Equations in R. Journal of Statistical Software, 45(3), 1-67. URL http://www.jstatsoft.org/v45/i03/ [↑](#footnote-ref-1)
2. Wood, S. and Scheipl, F. (2013). gamm4: Generalized additive mixed models using mgcv and lme4. R package version 0.2-1. Available at: http://CRAN.R-project.org/package=gamm4 [↑](#footnote-ref-2)
